# Comparative chromosome painting of pronghorn (*Antilocapra americana*) and saola (*Pseudoryx nghetinhensis*) karyotypes with human and dromedary camel probes

**DOI:** 10.1186/1471-2156-15-68

**Published:** 2014-06-12

**Authors:** Anastasia I Kulemzina, Polina L Perelman, Darya A Grafodatskaya, Trung T Nguyen, Mary Thompson, Melody E Roelke-Parker, Alexander S Graphodatsky

**Affiliations:** 1 Institute of Molecular and Cellular Biology, SB RAS and Novosibirsk State University, Novosibirsk, Russia; 2 Genetics and Genome Biology Program, Hospital for Sick Children, Toronto, ON, Canada; 3 Laboratory of Embryo Technology, Institute of Biotechnology, Vietnam Academy of Science and Technology, 18 Hoang Quoc Viet, Hanoi, Vietnam; 4 Institute of Animal Sciences, Swiss Federal Institute of Technology, (ETH), 8092 Zurich, Switzerland; 5 BSP-CCR Genetics Core, Center for Cancer Research, 21702 Frederick, MD, USA; 6 Laboratory of Animal Sciences Program, Leidos Biomedical Research, Inc., Frederick National Laboratory, 21702 Frederick, MD, USA; 7 Laboratory of Genomic Diversity, National Cancer Institute, 21702 Frederick, MD, USA

**Keywords:** Pronghorn, Antilocapra americana, Saola, Pseudoryx nghetinhensis, Comparative cytogenetics, Pecora, Phylogeny, Chromosome evolution

## Abstract

**Background:**

Pronghorn (Antilocapridae, 2n = 58) and saola (Bovidae, 2n = 50) are members of Pecora, a highly diversified group of even-toed hoofed mammals. Karyotypes of these species were not involved in chromosome painting studies despite their intriguing phylogenetic positions in Pecora.

**Results:**

To trace the chromosome evolution during very fast radiation of main families from the common Pecoran ancestor, high-resolution comparative chromosome maps of pronghorn and saola with human (HSA) and dromedary camel (CDR) painting probes were established. The human and dromedary camel painting probes revealed 50 and 64 conserved segments respectively in the pronghorn genome, while 51 and 63 conserved segments respectively in the saola genome. Integrative analysis with published comparative maps showed that inversions in chromosomes homologous to CDR19/35/19 (HSA 10/20/10), CDR12/34/12 (HSA12/22/12/22), CDR10/33/10 (HSA 11) are present in representatives of all five living Pecoran families. The pronghorn karyotype could have formed from a putative 2n = 58 Pecoran ancestral karyotype by one fission and one fusion and that the saola karyotype differs from the presumed 2n = 60 bovid ancestral karyotype (2n = 60) by five fusions.

**Conclusion:**

The establishment of high-resolution comparative maps for pronghorn and saola has shed some new insights into the putative ancestral karyotype, chromosomal evolution and phylogenic relationships in Pecora. No cytogenetic signature rearrangements were found that could unite the Antilocapridae with Giraffidae or with any other Pecoran families. Our data on the saola support a separate position of Pseudorigyna subtribe rather than its affinity to either Bovina or Bubalina, but the saola phylogenetic position within Bovidae remains unresolved.

## Background

The major group of hoofed mammals– Pecora includes five living families: Antilocapridae, Giraffidae, Moschidae, Cervidae, and Bovidae. This study is devoted to molecular cytogenetic investigation of karyotypes of two very interesting pecoran species belonging to Antilocapridae and Bovidae.

The family Antilocapridae appeared in North America and occupied the same ecological niche as Bovidae that evolved in the Old World. During the Miocene and Pliocene, they were a diverse and successful group, with many different species [1]. But now there is only one living species within this family – pronghorn (*Antilocapra americana*, AAM, 2n = 58), that is endemic in the deserts and dry grasslands of western North America [2]. The position of Antilocapridae on the Pecoran phylogenetic tree remains uncertain and there are two suggestions: the sister relationship to Giraffidae [3-5] or a basal position to other Pecoran families [4,6-10]. Cytogenetic studies of the pronghorn karyotype (2n = 58) revealed a high degree of similarity to the Pecoran ancestral karyotype (PAK, 2n = 58, Slate et al. [11]), having the same diploid number and the pronghorn X-chromosome likely being in the ancestral Pecoran state [12]. Therefore it was necessary to attempt to find some cytogenetic markers that could clarify the phylogenetic position of Antilocapridae within the Pecoran families. A combination of well described human and dromedary camel painting probes was used in this study, to establish the high resolution comparative chromosome map for pronghorn.

Recently, a new Bovidae species – saola (*Pseudoryx nghetinhensis*, PNG, 2n = 50) was discovered, living in the forest jungle, which separates Vietnam from Laos [13]. Molecular phylogenetic studies place this species in subfamily Bovinae, tribe Bovini. The position of saola within tribe Bovini remains uncertain, however most investigations suggested subtribal status Pseudoryina and a sister relationship with subtribe Bovina for this species [10,14,15]. Localization of nucleolar organizing regions (NORs) revealed the shared presence of three NORs in karyotypes of *Pseudoryx*, *Syncerus* and *Bubalus* suggesting the saola’s placement within the subtribe Bubalina [16]. Recent study of exon-primed intron-crossing (EPIC) autosomal loci indicate, however, that a sister-group relationship between Bovina and Bubalina, with Pseudoryna being basal, is the most strongly supported phylogenetic hypothesis [17]. The molecular cytogenetic study of saola karyotype by localization of cattle 29 Texas markers and three additional markers on saola chromosomes provided the first preliminary homology map for these two species [18]. Here we present the first genome-wide comparative map obtained by chromosome painting of saola karyotype with human and dromedary camel painting probes.

## Methods

Metaphase chromosomes of *Antilocapra americana* male were obtained from a cultured fibroblast cell line. The primary fibroblast cell line was established from the ear punch of the male *A. americana* (07 W11974/5, Worland, WY), provided by Wyoming Game and Fish Department, USA. The cell line of female *Pseudoryx nghetinhensis* was the same as used in the article of Nguyen et al. [19]. C-banding of *A. americana* chromosomes was carried out as described by Sumner [20]. The chromosomes of *A. americana* were arranged according to nomenclature given in the *Atlas of Mammalian Chromosomes*[21]. Chromosomes of *P. nghetinhensis* were arranged as previously described in the cytogenetic study of this species [18]. The same set of human and dromedary probes reported previously [22,23] has been hybridized to metaphase chromosomes of *A. americana* and *P. nghetinhensis* in this study.

The Zoo-FISH experiments were performed as described by Yang and Graphodatsky [24]. To achieve good spreading of the metaphases on the slide, the cell suspension was dropped on the wet cold slide, then slightly dried (until the suspension was completely spread on the slide), and then carefully washed twice by cold acetic acid - methanol (1:3 or 2:3) fixative. The thickness of the cytoplasm background covering the target metaphase chromosome was evaluated using phase-contrast on Axioscope 2 Plus microscope. G-banding on all metaphase chromosomes prior to FISH experiments was performed using the standard trypsin/Giemsa treatment procedure [25]. DOP-PCR amplified chromosome-specific DNA was labelled during the secondary PCR by incorporating biotin-16-dUTP or digoxigenin-dUTP. Biotin-labeled probes were visualized with avidin-FITC and anti-avidin-FITC (Vector Lab). Digoxigenin-labeled probes were detected by antidigoxigenin-DyLight549 (Jackson Immunoresearch). Images were captured and processed using Videotest 2.0 Image Analysis System and a Baumer Optronics CCD Camera mounted on Axioscope 2 Plus microscope (Carl Zeiss).

## Results

Human and dromedary painting probes were used to establish the genome-wide chromosome comparative map for the pronghorn and saola. All results are summarised in Table 1.

**Table 1:** Correspondence between conserved segments in Pecoran ancestral karyotype (PAK), pronghorn (AAM), saola (PNG), camel (CDR) and human (HSA)

**PAK**	**AAM**	**PNG**	**CDR**	**HSA**
A1	13 (3)	7	21/9/13	1
A2	1q (1)	1q	1	3/21
B1	3 (8)	4q	4/31	8/9
B2	2 (2)	6	5/13	2/1
C1	12 (11)	2q	15/28/4	2/9
C2	5 (5)	8	12/34	12/22
D	4 (4)	5q	7	7
E	7 (7)	9	3/22	5/19
F	6 (6)	3q	2	4
G	10 (10)	1p	6	14/15
H1	8 (9)	10	8	6
H2	17 (14)	11	25/29	8
I	18 (12)	2p	14	13
J	9 (13)	4p	19/35	10/20
K	16 (16)	13	23/21/13	1
L	15 (15)	12	10/33	11
M	26 (18)	5p	9	16/19
N1	24 (17)	14	2/32	4/12/22
N2	19 (19)	3p	16	17
O	14 (20)	15	3/22	5
P	21 (21)	16	6/27	14/15
Q	23 (22)	17	17	3
R	20 (23)	18	20	6
S	22 (24)	19	30/24	18
T	27 (25)	20	18	7/16
U	11 (26)	21, 23	11	10
V	28 (27)	22	26	4/8
W	25 (28)	24	10/33	11
X	X	X	X	X

The numbering of AAM chromosomes in parentheses corresponds to the nomenclature used by Cernohorska et al. [12].

### C-banding karyotype of pronghorn and chromosome painting on pronghorn chromosomes

**Figure 1:**
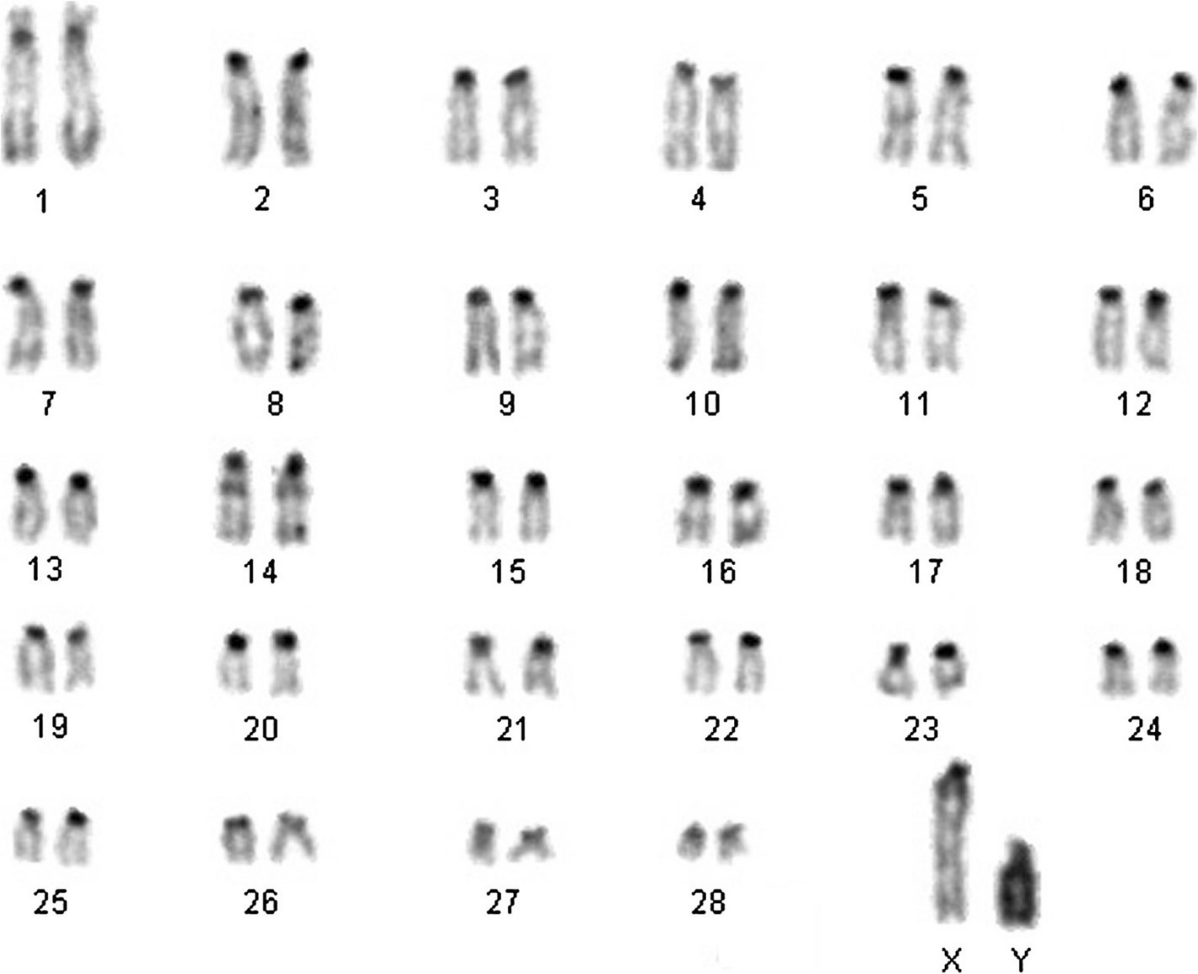
C-banded karyotype of pronghorn.

C-banding of pronghorn chromosomes (Figure 1) revealed that all autosomes of the pronghorn karyotype have centromeric constitutive heterochromatin blocks. Additionally there is an interstitial heterochromatic block on the chromosome AAM 14. The X-chromosome is represented by the largest acrocentric in the pronghorn karyotype, with a big heterochromatin region near the centromere. The Y-chromosome is a C-positive large acrocentric chromosome. C-banding of saola chromosomes was described previously [19].

**Figure 2:**
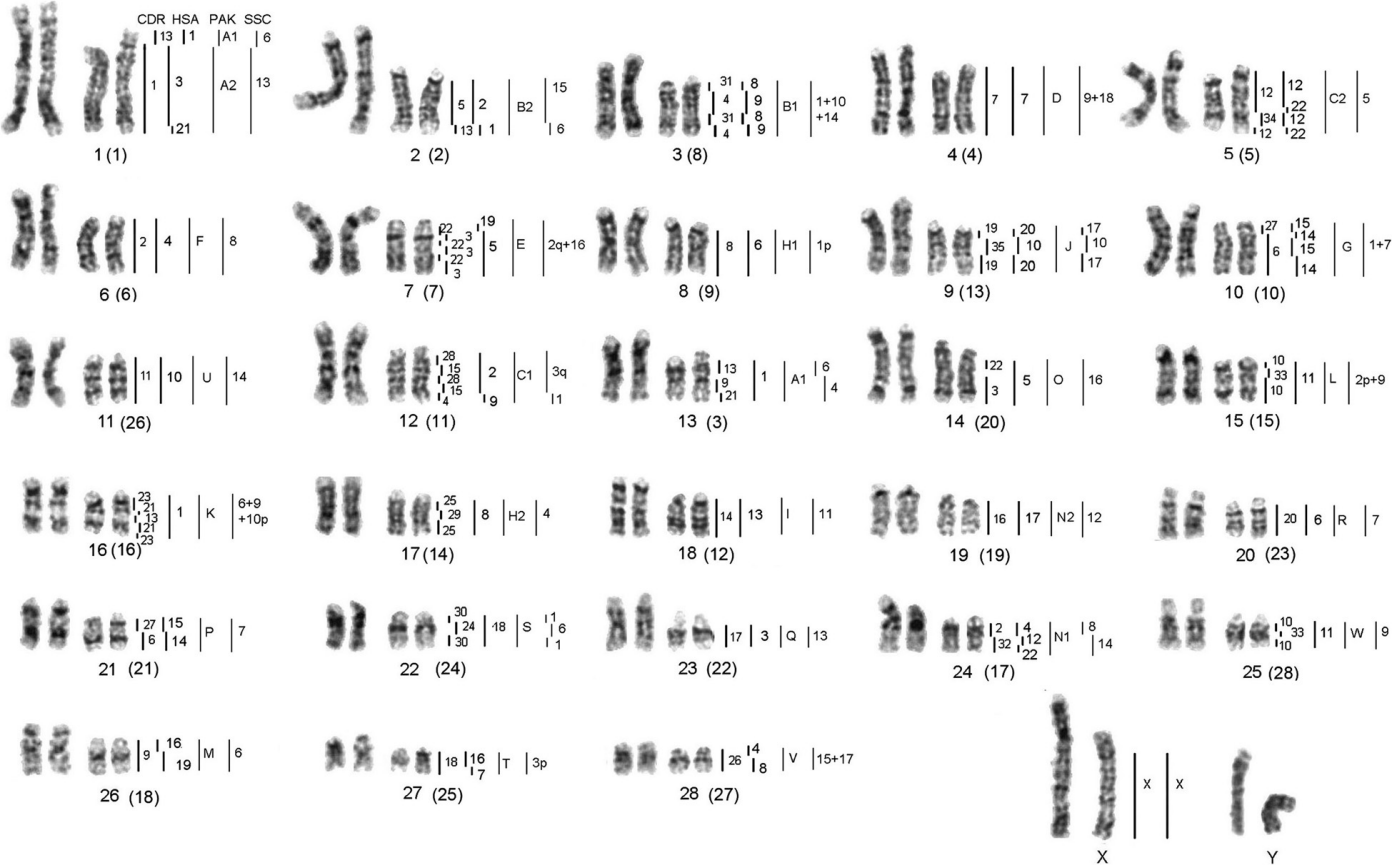
**G-banded karyotype of the pronghorn with homologies to human (HSA) and dromedary (CDR) painting probes.** AAM/SSC (*Sus scrofa*) homologies arebased on pig/cattle/human comparative map [23]. AAM/PAK (Pecoran Ancestral Karyotype) homologies are based on Slate et al. data [11]. The high resolution G-banding chromosomes are shown on the left.The pronghorn chromosome numbering used by Cernohorska et al. [12] is indicated in parentheses.

The painting probes from 22 human autosomes revealed 50 conserved segments on the pronghorn chromosomes (Figure 2), covering the whole genome except for two heterochromatic blocks: the interstitial on AAM 14 and pricentromeric region on AAM X. HSA probes 1 and 8 each painted four blocks in pronghorn karyotype. HSA 4, 9, 12, 14, 15 and 22 probes each revealed three conservative segments on pronghorn chromosomes. HSA 2, 3, 5, 6, 7, 10, 11, 16, 19 and 20 probes each detected two homologous segments. Human chromosomes 13, 17, 18, 21 each revealed one homologous block on pronghorn chromosomes, suggesting an *in toto* conservation of chromosomal synteny.

The painting probes from the 35 dromedary autosomes revealed 64 conserved segments on the pronghorn chromosomes (Figure 2). The CDR paints covered whole karyotype with the same two exceptions as described for human probes, due to the presence of heterochromatic blocks on AAM 14 and AAM X. We could not obtain any hybridization signal from the painting probe of the smallest camel chromosome CDR 36. Four conservative blocks (i.e. the highest number) in pronghorn karyotype were revealed with each of the dromedary chromosomes 3, 10, 13 and 22. Two dromedary autosomes 4 and 21 each detected three conservative blocks on pronghorn chromosomes. The dromedary chromosomes 2, 6, 9, 12, 15, 19, 23, 25, 27, 28, 30, 31 and 33 each revealed two homologous segments. The remaining 16 camel autosomes (i.e. 1, 5, 7, 8, 11, 14, 16, 17, 18, 20, 24, 26, 29, 32, 34 and 35) each detected one hybridization signal.

### Chromosome painting on saola chromosomes

**Figure 3:**
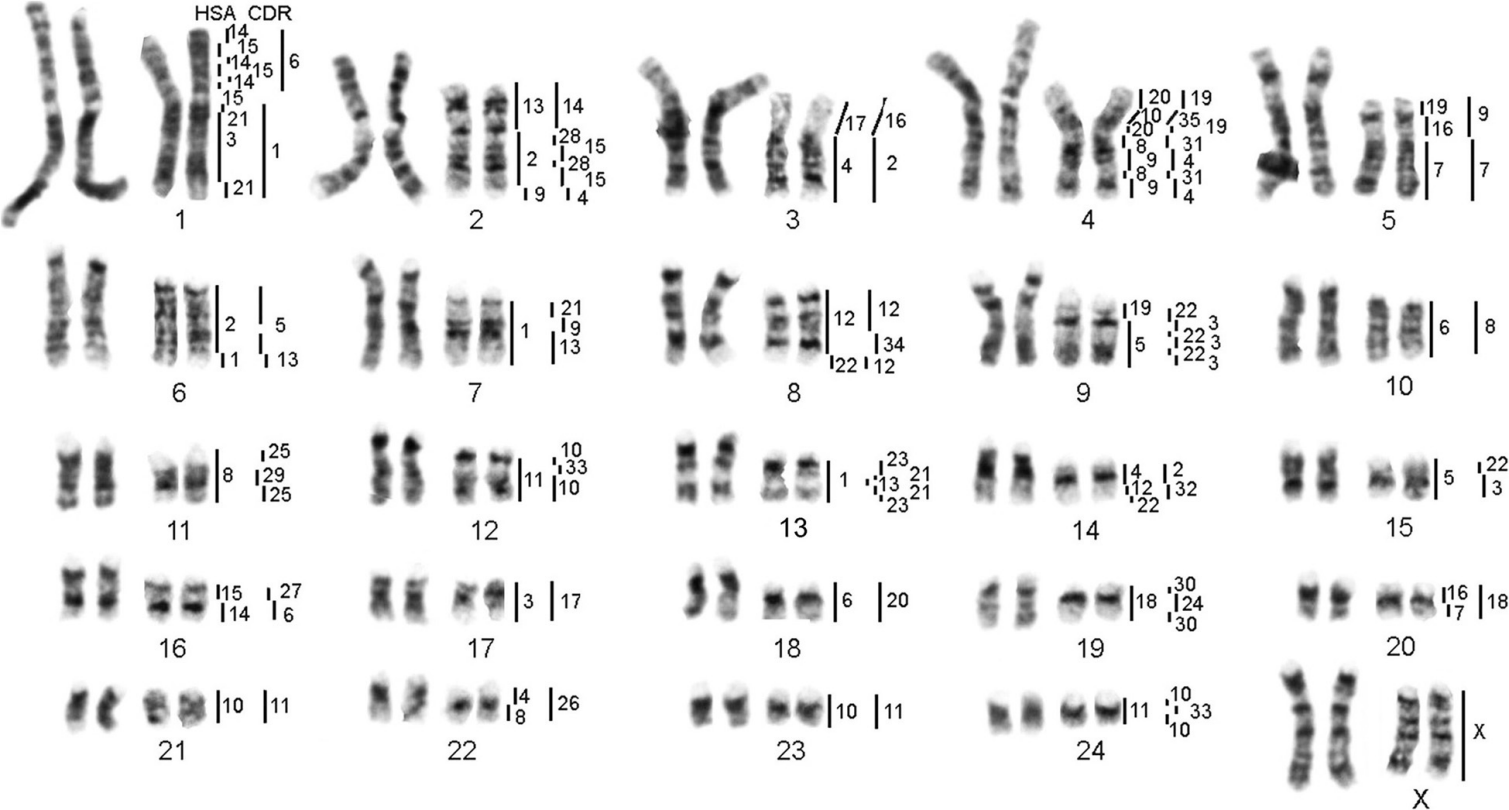
**G-banded karyotype of the saola with homologies to human (HSA) and dromedary (CDR) painting probes.** The high resolution G-banding chromosomes are shown on the left.

The painting probes from the 22 human autosomes revealed 51 conserved segments on the saola karyotype (Figure 3). The highest number (four segments) of conservative blocks was revealed with each of the probes HSA 8, 14 and 15. The probes HSA 1, 4, 9 and 10 each detected three homologous regions. The probes HSA 2, 3, 5, 6, 7, 11, 12, 16, 19, 20, 21 and 22 each delineated two homologous segments. The same human chromosomes 13, 17 and 18 each revealed one homologous block on saola chromosomes as in the pronghorn karyotype.

The 35 dromedary autosomal paints detected 63 homologous segments in the genome of the saola (Figure 3): three painting probes CDR3, 10, and 22 each revealed four homologous segment; CDR paints 4, 13, 21 each delineated three conservative segments; 13 paints (CDR 2, 6, 9, 11, 12, 15, 19, 23, 25, 28, 30, 21 and 33) detected two homologous regions; and 16 paints (CDR 1, 5, 7, 8, 14, 16, 17, 18, 20, 24, 26, 27, 29, 32, 34 and 35) each delineated one homologous segment. The probes containing CDR 36 again did not reveal any hybridization signal. The dromedary X chromosome probe painted the whole X chromosome of the saola.

## Discussion

It was shown previously that the combination of camel and human chromosome specific probes is a powerful tool for chromosome painting studies of Cetartiodactyla species and provides high resolution comparative chromosome maps [23,26-28]. Here we present genome-wide comparative maps for two Ruminantia species - pronghorn (Antilocapridae) and saola (Bovidae).

Our data on the pronghorn karyotype (Figure 2), obtained with human and camel chromosome specific probes, are generally in agreement with the results revealed by localization of cattle BAC-clones and microdissected probes [12]. The pronghorn karyotype differs from the Pecoran ancestral karyotype by one fission (on CDR13 homolog), one fusion (CDR1 + 13) and three inversion (CDR 19/35/19, CDR 12/34/12 (HSA 12/22/12/22), CDR 10/33/10). These inversions were found in karyotypes of some Pecoran families except Giraffidae [26,29]. The inversion in the conservative segment homologous to CDR 19/35/19 was described as specific synapomorphy that unites Cervidae, Bovidae and Moschidae families [26]. Inversion of CDR 12/34/12 (HSA 12/22/12/22) was revealed in cattle chromosome 5 [23] and Siberian musk deer chromosome 5 [26]. And inversion CDR 10/33/10 (conservative synteny homologous to Siberian roe deer chromosome 11) was shown as one of Cervidae karyotypes’ characteristic traits [28]. Thus we might propose a sister relationship of Antilocapridae and Cervidae families but this would contradict numerous molecular studies that place Antilocapridae as basal Pecoran families [9,10] or as sister taxon to Giraffidae [5,7,30].

**Figure 4:**
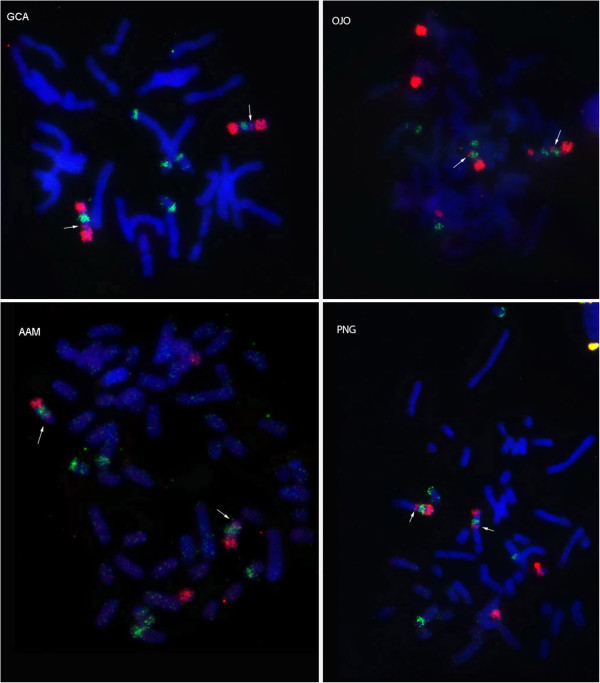
**FISH of dromedary camel (CDR 19 red and 35 green) painting probes onto: giraffe (GCA), okapi (OJO), pronghorn (AAM) and saola (PNG).** Arrows indicate revealed additional signals.

**Figure 5:**
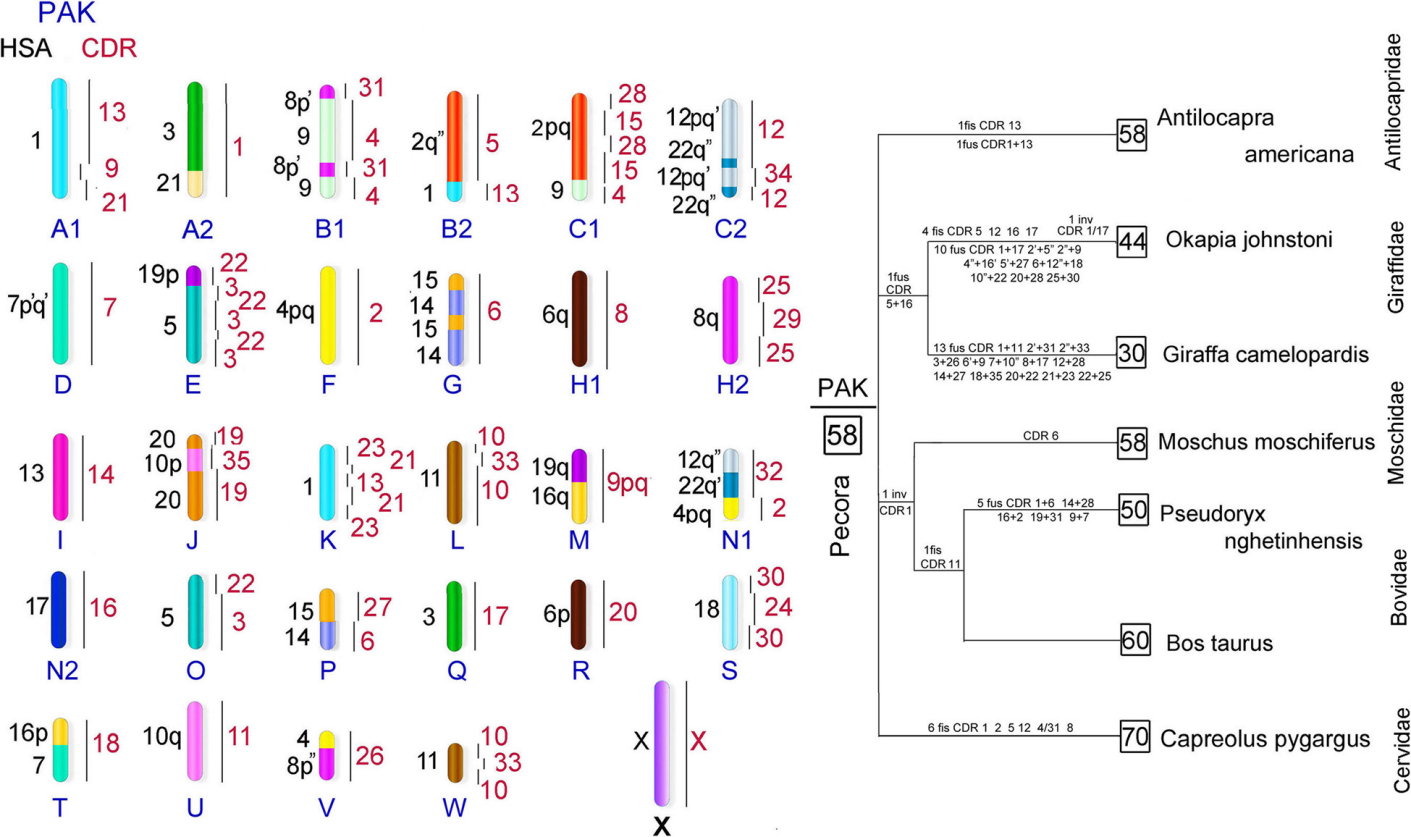
**Putative pecoran ancestral karyotype (PAK, 2n = 58) and a scenario of the karyotype evolution in Pecoran families.** Human (HSA) and dromedary (CDR) homologies are shown on the left and right of ancestral blocks. Chromosome rearrangements that formed karyotypes of pecoran species are given based on [23,26,28] and presented data. Numbers in squares indicate the diploid numbers.

Therefore we rechecked our localization of CDR 19, 35, 10, 33 and HSA 12, 22 chromosome specific probes on available Giraffidae, Bovidae and Moschidae species. The tiny signal from CDR 10 on the proximal part of homologous chromosome BTA 15, MMO 14, PNG 12, GCA 11q, OJO 20 and AAM 15 was revealed. The presence of the CDR10 signal indicates that inversion in CDR 10/33/10 is characteristic not only for Cervidae but also for other Pecoran families (Additional file 1: Figure S1). The additional small signal from CDR 19 was detected on proximal part of giraffe chromosome 13q and okapi chromosome 16 (Figure 4). Therefore the inversion CDR 19/35/19 is characteristic for Giraffidae too. Also we were able to obtain the additional signal from HSA 22 on chromosomes PNG 8, GCA 4q, OJO 15 and AAM 5 (Additional file 2: Figure S2). The finding of the inversions CDR 19/35/19, CDR 12/34/12 (HSA 12/22/12/22), CDR 10/33/10 in karyotypes of representatives of all Pecoran families makes these rearrangements characteristic for the Pecoran ancestral karyotype (Figure 5). Found inversions, however, do not change the ancestral Pecoran karyotype diploid number (2n = 58). Three additional inversions were identified that led to the formation of PAK from the Ruminantia ancestral karyotype. We conclude that the pronghorn karyotype was formed from the Pecoran ancestral karyotype by 1 fission (on CDR13 homolog) and 1 fusion (CDR1 + 13), and we have not revealed any cytogenetic markers that could confirm uniting Antilocapridae with Giraffidae or with any other Pecoran family.

Our comparative map obtained for saola karyotype with human and camel chromosome specific probes is in agreement with the results of FISH-mapping of 32 type-I markers [18] and is the first genome-wide comparison of saola karyotype. Saola karyotype has arisen from the Pecoran ancestral karyotype by 5 fusions (CDR 1 + 6, CDR 14 + 28, CDR 16 + 2, CDR 19 + 31, CDR 9 + 7), 1 fission (CDR 11) and 1 inversion (on CDR 1- HSA 3/21) (Figure 5). Inversion on CDR 1 homologous to HSA 3/21 was also found in the musk deer karyotype [12,26] and united the Moschidae and Bovidae families. Fission of a conservative segment homologous to CDR 11 (PNG 21, 23) is characteristic for Bovidae ancestral karyotype [23]. None of the remaining rearrangements unites saola with either river buffalo (*Bubalis bubalis*) [31] or gayal *(Bos frontalis)*[32]. Regarding other Bovidae subfamilies studied by chromosome painting, there are no common rearrangements of saola with Caprinae [31]. Thus chromosome rearrangement data indicate a separate position of Pseudorigyna subtribe rather than its affinity to either Bovina or Bubalina, leaving the saola phylogenetic position within Bovidae unresolved.

## Conclusion

The high resolution chromosome maps of pronghorn and saola obtained here represent complete comparative genome-wide maps that allow inclusion of these species into comparative genomic studies and the chromosome assignment of whole-genome sequences. High-resolution chromosome painting using a combination of dromedary camel and human probes has led to the identification of fine inversions in all Pecoran families (PAK chromosomes C2, J, L). The updated ancestral Pecoran karyotype (2n = 58) devised here is based now on the chromosome painting data from representatives of all five living Pecoran families. However, rapid speciation in Pecora that occurred over 30 million years [3] was not always accompanied by fixation of large-scale chromosome rearrangements. Chromosome rearrangement data rather support separate taxonomic status for Antilocaprinae (family) and Pseudoryina (subtribe) than affiliation with other taxa. Additional fine-scale mapping and sequence comparison are required to resolve the phylogenetic position of these two species in Pecora.

### Availability of supporting data

The data set supporting the results of this article are included within the article and its additional files.

## Abbreviations

AAM: Antilocapra americana; 2n: Diploid number of chromosomes; BAC: Bacterial artificial chromosome; BTA: Bos taurus; CDR: Camelus dromedarius; DOP-PCR: Polymerase chain reaction using degenerated oligonucleotide primers; FISH: Fluorescence in situ hybridisation; GCA: Giraffa camelopardalis; GTG: Banding G banding by trypsin using Giemsa; HSA: Homo sapiens; MMO: Moschus moschiferus; OJO: Okapia johnstoni; PAK: Pecoran ancestral karyotype; PNG: Pseudoryx nghetinhensis.

## Competing interests

The authors declare that they have no competing interests.

## Authors’ contributions

KAI designed and performed comparative chromosomal painting experiments, analyzed the data and wrote the manuscript. PLP have carried out cell culture work and edited manuscript critically for important intellectual content. MT performed the primary cell culture work with pronghorn sample. MRP participated in discussion of the study and facilitated pronghorn sample collection. GAD and NTT have performed cell culture work with saola sample. ASG conceived the study, revised the manuscript and gave final approval of the version to be published. All authors read and approved the final manuscript.

## Supplementary Material

Additional file 1: Figure S1 FISH of dromedary camel (CDR 10 red and 33 green) painting probes onto: giraffe (GCA), okapi (OJO), pronghorn (AAM), saola (PNG), Siberian roe deer (MMO) and cow (BTA). Arrows indicate revealed additional signals. Click here for file

Additional file 2: Figure S2 FISH of human (HSA 12 green and 22 red) painting probes onto: giraffe (GCA), okapi (OJO), pronghorn (AAM) and saola (PNG). Arrows indicate revealed additional signals. Click here for file
