# Supplementary figures and images for: Comparative chromosome painting of pronghorn (Antilocapra americana) and saola (Pseudoryx nghetinhensis) karyotypes with human and dromedary camel probes
Source: BMC Genet. 2014 Jun 12;15:68. doi: 10.1186/1471-2156-15-68 (PMC4061535; doi:10.1186/1471-2156-15-68)

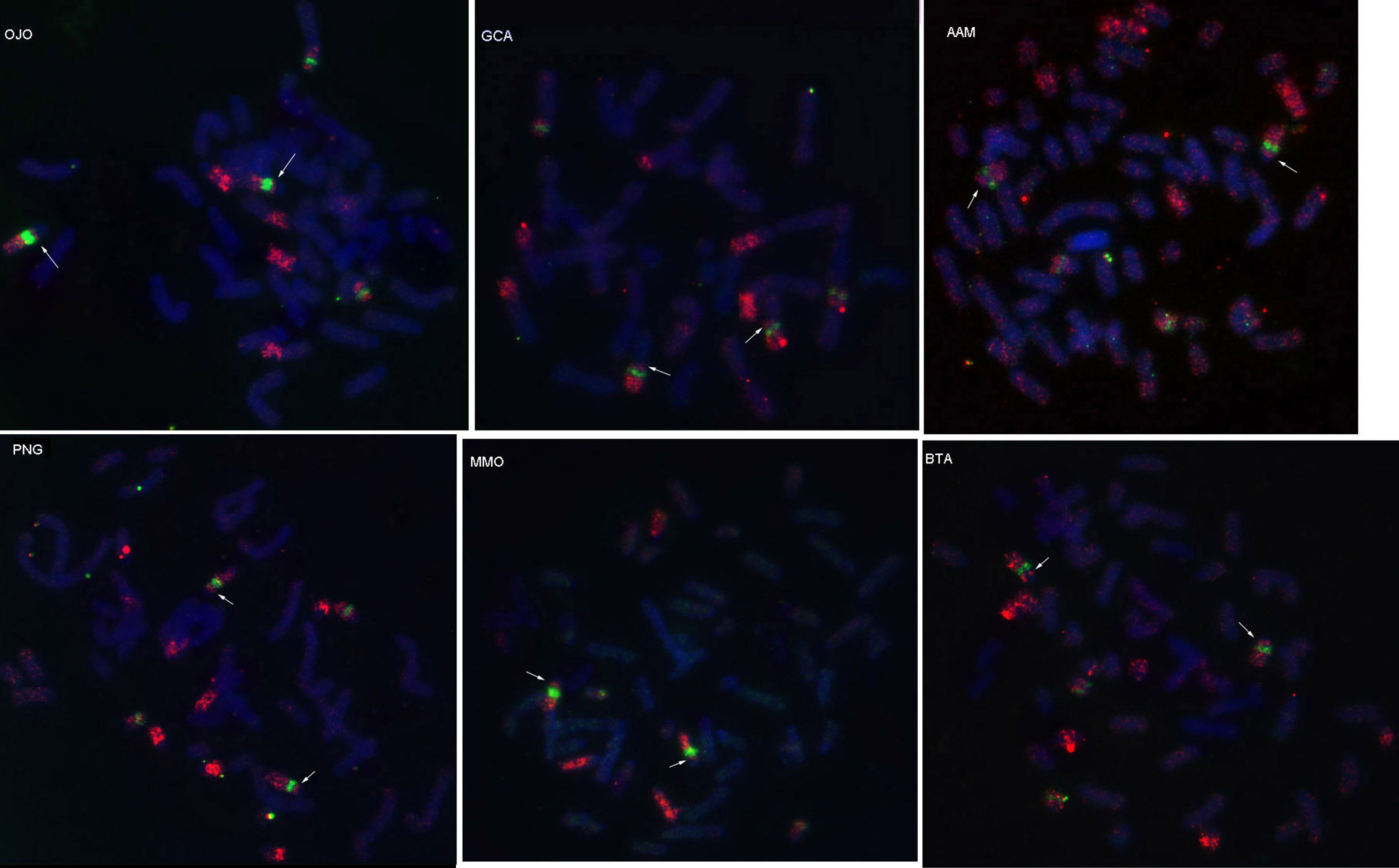

Supplement: Additional file 1: Figure S1 — FISH of dromedary camel (CDR 10 red and 33 green) painting probes onto: giraffe (GCA), okapi (OJO), pronghorn (AAM), saola (PNG), Siberian roe deer (MMO) and cow (BTA). Arrows indicate revealed additional signals. [file 1471-2156-15-68-S1.jpeg]

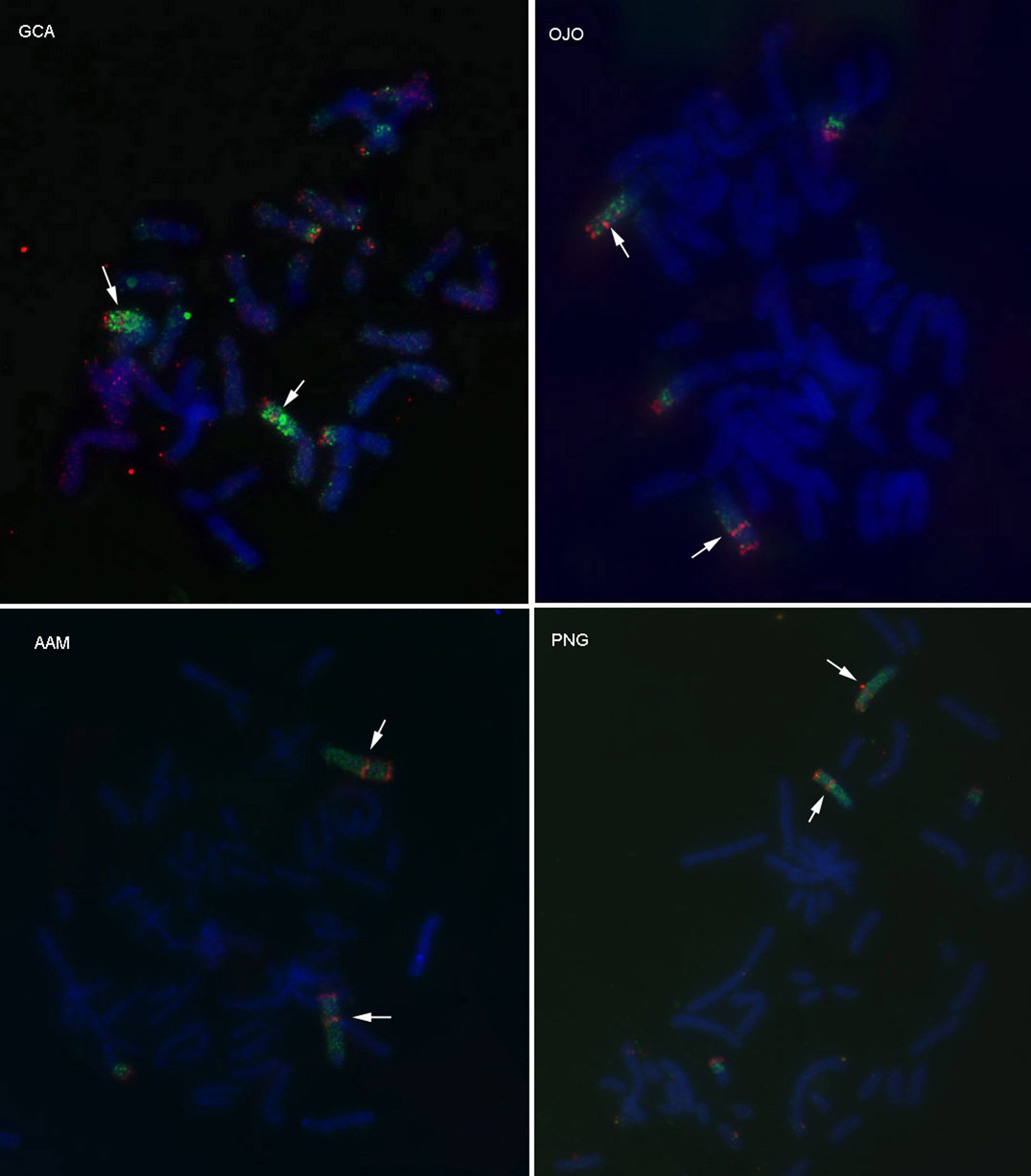

Supplement: Additional file 2: Figure S2 — FISH of human (HSA 12 green and 22 red) painting probes onto: giraffe (GCA), okapi (OJO), pronghorn (AAM) and saola (PNG). Arrows indicate revealed additional signals. [file 1471-2156-15-68-S2.jpeg]
